# Sculpting tissues by phase transitions

**DOI:** 10.1038/s41467-022-28151-9

**Published:** 2022-02-03

**Authors:** Pierre-François Lenne, Vikas Trivedi

**Affiliations:** 1 Aix Marseille Univ, CNRS, UMR 7288, IBDM, Turing Center for Living Systems, Marseille, France; 2 European Molecular Biology Laboratory (EMBL), Barcelona, 08003 Spain; 3 EMBL Heidelberg, Developmental Biology Unit, Heidelberg, 69117 Germany

**Keywords:** Biophysics, Developmental biology, Systems biology, Phase transitions and critical phenomena

## Abstract

Biological systems display a rich phenomenology of states that resemble the physical states of matter - solid, liquid and gas. These phases result from the interactions between the microscopic constituent components - the cells - that manifest in macroscopic properties such as fluidity, rigidity and resistance to changes in shape and volume. Looked at from such a perspective, phase transitions from a rigid to a flowing state or vice versa define much of what happens in many biological processes especially during early development and diseases such as cancer. Additionally, collectively moving confluent cells can also lead to kinematic phase transitions in biological systems similar to multi-particle systems where the particles can interact and show sub-populations characterised by specific velocities. In this Perspective we discuss the similarities and limitations of the analogy between biological and inert physical systems both from theoretical perspective as well as experimental evidence in biological systems. In understanding such transitions, it is crucial to acknowledge that the macroscopic properties of biological materials and their modifications result from the complex interplay between the microscopic properties of cells including growth or death, neighbour interactions and secretion of matrix, phenomena unique to biological systems. Detecting phase transitions in vivo is technically difficult. We present emerging approaches that address this challenge and may guide our understanding of the organization and macroscopic behaviour of biological tissues.

## Abstract (web summary)

Many biological processes require changes in the physical properties of cells and their surroundings. Here, Lenne and Trivedi discuss recent findings in biological systems in terms of phase transitions in inert physical systems from both theoretical and experimental perspectives.

## Phase transitions in tissue morphogenesis

Biological tissues require the appropriate organization of their constituent components—the cells—needed for maintaining proper structure and function. Similar to any multicomponent system, interactions between the constituent components largely dictate the overall behavior or the macroscopic state. For instance, the three states of matter—solid, liquid, and gas—are characterized by distinct interactions between its component particles at the microscopic level that manifests in macroscopic properties such as fluidity, rigidity, resistance to changes in shape and volume.

**Fig. 1:**
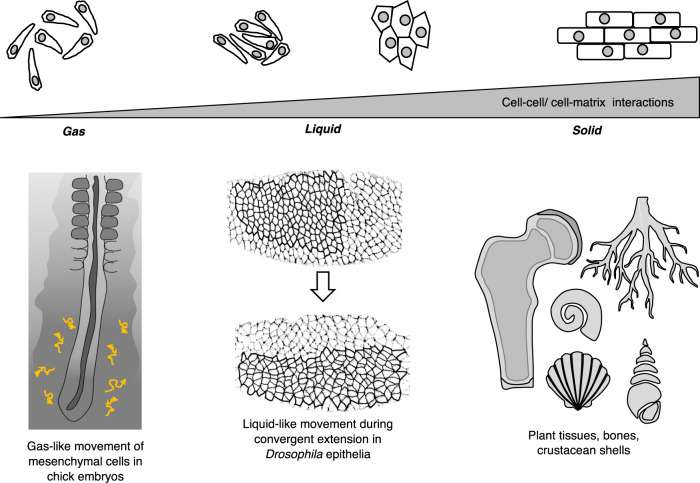
Rich phenomenology of states of matter displayed by biological materials. Similar to physical matter, biological materials display both fluid-like and solid-like properties at the population level, resulting from the interaction of cells with their neighbors and the extracellular media. State of tissue can be gas-like (e.g., mesenchymal cells in chick embryos, adapted from ref. ^21^ with permission from *Nature*), liquid-like (e.g., epithelial tissue during gastrulation in *Drosophila*, adapted from ref. ^112^ with permission from *Nature Cell Biology*) or solid-like (e.g., bones, cartilage, tree barks).

Biological systems also display a rich phenomenology of such states of matter that result from the interaction of cells with their neighbors and the extracellular media, which is also often created by the cells themselves (Fig. 1). For example, bones, cartilage, or tree barks are examples of solid-like materials seen in biology. Fluid-like behavior is generally more commonly observed in biological tissues, especially within the animal kingdom. Particularly during the development of embryos whose shape changes are due to an interplay between individual cell shape changes and cell topological rearrangements—for example in gastrulation-embryonic tissues behave like liquids that cannot strongly resist changes in shape. Some epithelial tissues exhibit the characteristics of fluid phases with a high degree of long-range orientational order, like liquid crystals^1^. In some cases, it is justified to consider the state of tissues as gas-like where interactions between individual cells are minimal and their movements are analogous to those for gas molecules (Fig. 1). Plant cells, on the other hand, owing to their saturating turgor pressure from vacuoles within a cellulose-based cell wall display more “permanent” solid-like behavior in collectives (e.g., plant roots, plant vasculature). However, a true solid-like behavior, distinguished by resistance to shape and volume changes, is often displayed in scenarios when the cells are completely replaced by their own extracellular matrix (ECM) or mineral secretions over time (such as in crustacean or insect shells, bone or tree barks, orcartilage) (Fig. 1).

The specific molecular makeup and neighbor arrangements result in different supracellular properties in cohorts of cells for both epithelial and mesenchymal types and as a result either can behave like solid or fluid. Generally, strong adhesiveness and tight packing of cells are achieved in the epithelial state and large-scale cellular rearrangements characterize loosely packed mesenchymal cells. Yet a collection of mesenchymal cells can still be confined within a small region despite large movements at the individual level and therefore result in the overall structural stability of the region. Evidence of such a fluid-to-solid transition in mesenchyme was recently elucidated in the context of the gradual “solidification” of tissue in the zebrafish mesodermal progenitor zone as cells move into presomitic mesoderm, where they are more packed^2^. In this case, the cell density affects the local tissue stiffness, and thus controls the solidification process. In confluent tissues where cell density is constant, solidification results from changes in cell–cell adhesion and cortical tension^3^ or in cell–cell and cell–substrate adhesion^4^. Similarly, a tightly packed epithelium can be “fluid-like” with its cells rearranging and/or moving collectively^5^. Different phases can coexist in a tissue; for example, cells can form fluid-like clusters that are exchanging cells with a gas-like phase. Such coexistence of cellular phases is observed in vitro^6^ and for tumors where individual (gas-like) cells emanate ("evaporate") from a fluid or even solid-like tumor. Cell clusters behaving like fluid droplets are able to migrate from a primary tumor and disseminate while maintaining their epithelial character^7^.

Unlike most inert physical systems, biological systems are characterized by growth and changes in material properties of its constituent cells at different timescales. For example, a population of cells can change its macroscopic (supracellular) behavior by changes in its microscopic (intracellular) properties such as specific cell surface or membrane proteins, rearrangement of intracellular cytoskeleton, changes in the number, size, and distribution of internal organelles such as vacuoles in plant cells. As a result, the state of a tissue is often transitory and the tissue can change its state in a rapid or slow manner depending upon the molecular processes involved. Epithelial to mesenchymal transition in animal cells is one such example and the timescale of this can range from a few minutes (zebrafish gastrulation) to several hours (mouse gastrulation) to several days (cancer). Zebrafish blastoderm has been shown to change its state from a more solid-like to fluid-like on a one-hour timescale by modulating the timescale of its cell–cell contacts^8^.

It seems that such transitions from a rigid to a flowing state, which can be conceptualized as the tissue’s solid-like and liquid-like phases, and vice versa define much of what happens in many biological processes especially during tumorigenesis and morphogenesis. It is important to note that while a biological tissue can change its hitherto mentioned transitory state by changes at the molecular level, mixed solid and fluid-like behavior is also an inevitable consequence of the fact that most cellular systems are viscoelastic (Box 1). Similar to inert viscoelastic materials, biological tissues also display a solid-like behavior at a shorter timescale and viscous fluid-like behavior at longer timescales^9,10^. It is possible that this viscoelastic timescale itself can change dramatically over time depending upon the molecular composition of the tissue and such changes can amount to a phase transition over the relevant timescale. However, as we will describe next, changes between solid to fluid behavior over a given viscoelastic timescale are not true phase transitions (Box 1). It is therefore crucial to consider the timescale of the process when analyzing changes in a biological system from a particular state (rigid or fluid-like) to another.

The hallmark of a phase transition is a change in the order of the system (Box 1). Such changes in properties can be abrupt (i.e., discontinuous, first-order phase transitions) or gradual (i.e., continuous, second-order phase transitions). For example, liquid to solid transition in water results in an abrupt change in the organization of water molecules as a periodic lattice whereas ferromagnetic materials are known to display gradual changes in the internal order. Such changes—abrupt or gradual—typically result from gradual changes in external conditions (control parameters) such as temperature, pressure, or density. In biological systems, though, changes in conditions are often internal such as growth rate, cell division, migration, adhesion, arrangement, etc. Unlike in a true fluid-to-solid transition where a spontaneous emergence of long-range crystalline order occurs, the rigid phase transitions in biological systems are characterized by the persistence of a disordered state of matter both in the solid and liquid states. Such phase transitions, known as jamming (Box  2), have been discussed extensively for inert materials such as foams, emulsions, granular materials, and glasses. Motivated by the work done in physics and engineering, jamming has turned out to be an effective paradigm for conceptualizing the emergence of rigidity in biological tissues in both 2D and 3D contexts^2,4,11–19^, whereby crowding, tension-driven rigidity, and reduction of fluctuations—all three mechanisms individually or simultaneously—can arrest cell motion resulting in a “jammed tissue”^20^. Analogous to multi-particle systems where the particles can interact with each other and show sub-populations characterized by specific velocities, biological systems can also display kinetic phase transitions (Box 1) and jamming in collectively moving confluent cells.

In this Perspective, we will discuss the above-mentioned phase transitions, both from theoretical perspective as well as experimental evidence in biological systems. In particular, we will discuss what the concepts of ordered and disordered systems (Box 1) mean in the biological context, what are the main physical determinants for fluid-to-solid transitions in biological tissues which would play a role analogous to stress, density, and especially temperature in the conventional matter and which criteria determine such phase transitions in tissues. We will then present approaches to detect such phase transitions in situ. Toward the end, we will also speculate about molecular realizations of fluid/solid transitions and critically discuss the opportunities and limitations of the analogy between collective effects in living systems and conventional phase transitions.

Box 1 Box definitions 1 **Viscoelasticity** Viscoelasticity is made up of two words, viscosity, and elasticity, and denotes the behavior of materials, such as polymers and biological materials, that behave neither as pure fluids nor pure solids, but possess both viscous and elastic properties. The mechanical response of such materials is dependent upon how quickly the load is applied or removed. Most biological materials are predominantly elastic at short timescales (on the order of tens of seconds to minutes) and viscous at long timescales (on the order of tens of minutes to hours).

**(Thermodynamic) phase transition** The term phase transition describes the transition between different states of matter such as solid, liquid, or gaseous states. A phase is characterized by physical properties (density, order), which are homogeneous in thermodynamic systems. Phase transitions usually result from a change of a control parameter, such as pressure or temperature. A classical example is the solidification of liquid water into crystalline ice when the temperature goes below 0 °C at atmospheric pressure. In the solidification process, translation symmetry is broken and water molecules organize in a periodic lattice. A long-range crystalline order rarely develops in multicellular systems, except in specific organs such as the compound eye of insects, and the analogy to liquid/crystal transition seems not to be adequate to describe living systems. A more relevant type of transition is the jamming transition (Box 2) that is observed for a large class of materials including granular media, colloidal suspensions, pastes, foams, and glass-forming liquids.

**Kinetic phase transition** A system consisting of self-propelled components can undergo a change from a random (disordered) motion of the components to aligned (large-scale ordered) motion, depending upon noise (in the velocity) and density. In analogy to true thermodynamic phase transitions, such changes in the kinetic behavior of a system when the control parameter passes through a critical point are termed “kinetic phase transition” (also referred to as flocking phase transition). For example, cells, such as keratocytes, that migrate as individuals at low cell density form coherent groups of motile cells, when the cell density exceeds a critical value; they exhibit a transition from a disordered to ordered state^33^. Similar transitions are observed in flocks of birds and schools of fish (for a comprehensive review, see ref. ^104^).

**Ordered system** The states of matter exhibit different structural orders: crystalline solids show perfect order, their constituent molecules being arranged in periodic lattices, while liquids lack spatial order. Phase transitions are generally characterized by changes in orders; for example, the liquid to solid transition is accompanied by the change of structural order. However, glasses are disordered materials that lack the periodicity of crystals but behave mechanically like solids. The common way to form a glass from a liquid is to cool it fast enough to prevent crystallization, thus ordering is not completed. The order/disorder can be quantified by studying the spatial correlations of density and orientation. Glasses are disordered like liquids and behave mechanically like solids.

## Origins of phase transitions: molecular, cellular, and multicellular

**Fig. 2:**
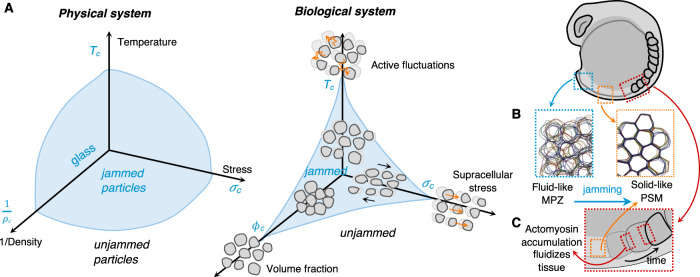
Jamming transition in biological materials. **A** Physical systems under the influence of external conditions such as temperature, pressure, or density can undergo flowing to rigid phase transitions known as jamming, without the spontaneous emergence of long-range order. An analogous phase diagram in biological systems has three control parameters: active fluctuations, supracellular stress, and volume fraction (adapted from ref. ^2^ with permission from *Nature*). **B** During axial elongation in zebrafish, cells leaving the mesodermal progenitor zone (MPZ, blue) to mature into presomitic mesoderm (PSM, orange) undergo a jamming transition. Within MPZ high cell–cell contact length fluctuations (high effective temperature) and more extracellular space renders the tissue fluid-like, as compared to the PSM where cell rearrangements and cell mixing is halted due to smaller extracellular spaces and low cell–cell contact fluctuations (low effective temperature). This jamming of the tissue acts as a rigid support biasing tissue expansion (elongation) toward the posterior direction (adapted from refs. ^2,41^ with permission from *Nature* and *Nature Physics*). **C** Progressive accumulation of F-actin and myosin II at the posterior boundary of younger somites leads to a local increase in tension fluctuations that transiently fluidizes the tissue for remodeling. Once somites are physically pinched off due to this boundary tension increase and fluidization, tissue returns to its rigid state maintaining the shape of somites (adapted from ref. ^40^).

Phase transitions in inert physical systems are controlled by a limited number of (control) parameters, such as temperature, volume, and stress (Box 1, Fig. 2). Given the complexity of biological systems, it can be expected that this small number will be replaced by a much larger one, thus transforming simple phase diagrams into complex multidimensional ones. Potential control parameters can be biochemical (pH, O_2_, signaling molecules, etc.) or physical (pressure, density, etc.), acting from the molecular to the multicellular scale. Despite this complexity, it is tempting to adapt the standard liquid–solid phase diagram to multicellular systems by replacing the usual control parameters by their potential cellular counterparts. For example, in cell monolayers undergoing a transition that resembles the jamming transition (Box 2), it was proposed to replace the temperature by cell motility, volume fraction by the density, and stress by the inverse of cell–cell adhesion (Fig. 2)^18^. These parameters have molecular origins and can result in changes at the cellular and multicellular levels leading to phase transitions. In this section, we will discuss instances and sources of phase transitions and probe into biological origins of such solid–fluid transitions in development and disease.

### Changes due to cellular motility

Unlike inert systems, phase transitions in living systems can be triggered by changes in the constituents’ dynamics and interactions. Cellular motility and cellular rearrangements are central in these transitions.

#### Changes due to cellular motion

One of the most striking changes in the morphology of early embryos is the extension of the body shape along the anteroposterior (A–P) axis. In vertebrates, body extension involves basic cellular behaviors: cell migration, cell proliferation, and cell rearrangements. In the chick, posterior elongation mainly results from proliferation and a graded random motility of cells in the presomitic mesoderm (PSM)^21^. The graded random motility in anterior-to-posterior provides a directional bias in the elongation toward the posterior part of the embryo. Analysis of cell movement in the PSM revealed that mesenchymal cells exhibit a Brownian motion relative to the underlying extracellular matrix, suggesting a gas-like behavior (Fig. 1A).

Comparing cells with fluid molecules, it is tempting to construct an analogy between single-cell diffusion within the tissue and thermal diffusion in Brownian systems^21^, and thus construct an effective temperature, which depends on the diffusion constant. This example shows that during development, a tissue can change its fluidity through a change in the motility of its constituent cells. However, further research in this direction may help us to understand how such biological processes can be conceptualized as phase transition phenomena.

#### Changes due to actively driven cellular rearrangements

**Fig. 3:**
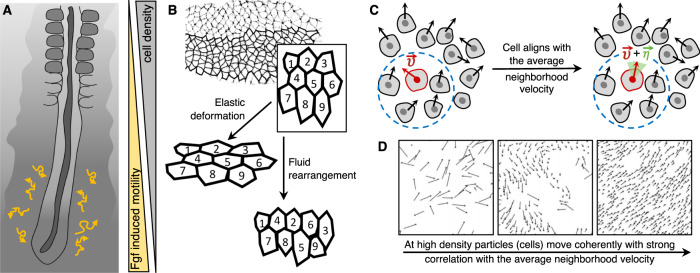
Phase transitions resulting from changes in cellular motility. **A** In chick presomitic mesoderm (PSM), mesenchymal cells display Brownian motion relative to the underlying extracellular matrix, such that the overall tissue has a fluid-like behavior. They undergo gradual “solidification” as they move from posterior to anterior under the influence of Fgf (adapted from ref. ^21^ with permission from *Nature*). **B** Active cellular rearrangements, due to myosin-dependent junction remodeling, during germband elongation of the *Drosophila* embryo render the tissue fluid-like due to neighbor exchanges (adapted from ref. ^112^ with permission from *Nature Cell Biology*). On the contrary, an elastic deformation keeps the same configuration between neighbors. **C**, **D** Kinetic phase transition of motile cells marks the emergence of self-ordered motion in systems of self-propelled particles. Within a collection of motile cells, each cell can change its direction of motion ($\overrightarrow{{{{{{{{\boldsymbol{\nu }}}}}}}}}$), with some perturbation ($\overrightarrow{{{{{{{{\boldsymbol{\eta }}}}}}}}}$ denoted by green shade) depending upon the average direction of motion in its neighborhood (denoted by blue circle). This leads to progressively increasing correlation of velocities as a function of density and noise the particles move with, resulting in a novel phase transition from no transport to finite net transport through spontaneous symmetry breaking of the rotational symmetry (adapted from refs. ^31,33^ with permission from *Physical Review Letters and Physical Review E*).

Fluid materials are characterized by particles rearranging irreversibly with each other when submitted to shear. In tissues, cellular rearrangements can be triggered by active processes such as contractions of the actomyosin network^22^ and have been shown to cause fluidization of epithelial tissues^23^. A classical example of cellular rearrangements that contribute to embryo shaping is the germband elongation of the *Drosophila* embryo^22,24^. The cellular rearrangements also called cell intercalation, involve anisotropic remodeling of cell junctions powered by contractile actomyosin networks. They participate in the fluidity of the tissue that would otherwise deform elastically (Fig. 3B).

In the chick embryo, fluidity emerges from changes in cell motility but also as a consequence of cell division. Cell division actively promotes cell rearrangements: daughter cells separate from each other after dividing and remodel their junctions with cells in the vicinity^25^. These local rearrangements are required for proper gastrulation patterning and if cell division is inhibited, the epithelial organization appears stabilized. At the molecular level, low actomyosin contractility facilitates junction remodeling during gastrulation, while at an earlier stage, high levels of actomyosin seem to prevent cell division-mediated rearrangements. These observations are consistent with a theoretical model that predicts that cell division (and apoptosis) occurring in elastic tissues introduce a dynamic reorganization that tends to a fluid-like behavior with well-defined shear and bulk viscosities^26^.

Actively driven cellular rearrangements lead to shape changes and flows at the tissue scale. There is increasing evidence that the boundary conditions for a given pattern of cellular rearrangements affect the macroscopic changes. In the *Drosophila* genitalia apparatus whose boundaries are circular, anisotropic rearrangements of epithelial cells lead to unidirectional rotation^27^. This example emphasizes the coupling between local mechanics and shape.

### Changes due to cell density

The cell density or equivalently the volume fraction is a physical parameter relevant to phase transition, as it largely determines the frequency of the interactions between particles or cells. In the elongating tail of the zebrafish embryo, the volume of extracellular space decreases towards the posterior and reaches a value below which the cellular material displays a significant yield stress preventing cell rearrangements^2^. This fluid-to-solid transition is similar to what happens in aqueous foams when the free volume fraction is below 0.36^28^. Mongera et al. showed that the gradients in yield stress and in the volume of extracellular space are controlled by the cell-adhesion protein N-cadherin (although the concentration of the protein is not itself graded).

**Fig. 4:**
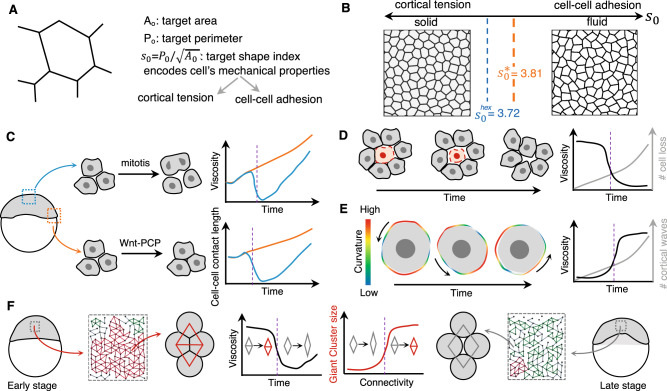
Phase transitions resulting from dynamic modulation of cellular contacts. **A**, **B** A balance between cortical tension and strength of cell–cell adhesion in non-motile cells can lead to solid–fluid transition in a density-independent manner. The control parameter (shape index *s*_0_) determines the probability of local cellular rearrangements. In an epithelium, the shape index corresponding to those for regular hexagons (3.72) marks the loss of stability and the rigidity transition occurs at a value of shape index that corresponds to regular pentagons (3.81, adapted from ref. ^3^ with permission from *Physical Review X*). **C** Cell divisions can lead to fluidization of tissues due to the loss of cell–cell contacts during mitotic cell rounding. In early zebrafish embryos (4 h post fertilization), cells in the blastoderm center undergo mitosis showing a decrease in cell–cell contact length and increase in interstitial gaps thereby leading to a temporary decrease in tissue viscosity and fluid-like behavior. However, the cells at the margin, do not show fluidization due to noncanonical Wnt signaling that strengthens cell–cell contacts (adapted from ref. ^8^ with permission from *The Embo Journal*). **D** Loss of cells in densely packed tissue (due to cell death or delamination) can lead to loss of cell–cell junctions and reduction in tissue viscosity and leading to fluid-like behavior (adapted from ref. ^46^ with permission from *Nature*). **E** Pulsed contractions of cells due to actomyosin contractility (such as ones seen in early mouse embryos at the eight-cell stage) when suppressed in tissues, due to cell–cell contacts, result in forces at the long timescale and increase the overall mechanical integrity of the tissue (adapted from ref. ^50^ with permission from *Nature Cell Biology*). **F** Changes in adhesion-dependent cell connectivity (such as ones in zebrafish blastoderm) around a critical value can lead to rigidity phase transition as predicted by percolation theory. The sudden disappearance of giant rigid clusters (red) below a critical value of cell connectivity can lead to an abrupt decrease in tissue viscosity making the tissue more fluid-like (adapted from ref. ^56^ with permission from *Cell*). Dashed vertical lines (purple) in the graphs in **C**–**F** denote critical points (in relevant parameter space) where phase transitions occur.

*Is the density a control parameter relevant to biological tissues?* Epithelial tissues are made of packed cells, a situation that corresponds to a volume fraction close to one. As both jammed and unjammed states are observed in such crowded systems, it is reasonable to question the use of density as a control parameter^3,4,16^. Early models of epithelial mechanics^29^ identified the existence of mechanically distinct networks that are reminiscent of jammed and unjammed states: a solid-like hexagonal network and a liquid-like soft network. Hexagonal networks have both a bulk and a shear modulus. In contrast, soft networks have vanishing shear modulus and behave more like a liquid in which cells can move and past one another easily. In such models, the mechanical energy of cells in the network is the sum of three terms: a term that is associated with the cell area compressibility, a term that is associated with the cell perimeter elasticity attributable to the cell stiffness and a term that is associated to the contact line energy attributable to adhesion and cortical tension. Changes in cell shapes are constrained by parameters that are the preferred perimeter P0 and area A0 that cells want to acquire. In this transition, the tissue can solidify if the cells decrease their preferred perimeter P0 relative to their preferred area A0. The control parameter of the jamming/unjamming transition is the ratio between the observed perimeter and the square root of the observed area (Fig. 4A). It is called the shape index. For a solid tissue, the shape index is precisely 3.81 (which is the perimeter-to-surface ratio of a perfect pentagon). If this index exceeds 3.81, the tissue is more fluid-like. This prediction is realized in epithelial monolayers cultures from lungs of human patients^16^ that exhibit jamming transitions. In cultures of cells derived from asthmatic donors compared with those from non-asthmatic donors, jamming transitions is delayed. It suggests that maintenance of fluidity of epithelial layers might contribute to pathogenesis, while jamming might be a functional state for epithelium to act as a barrier. Recent work in *Drosophila* germband epithelium has evaluated the influence of cell packing on the shape index and has shown that it can actually vary between 3.72 and at least 3.9 depending how many pentagons and how many manyfold vertices are present in a tightly packed epithelium^30^.

**Fig. 5:**
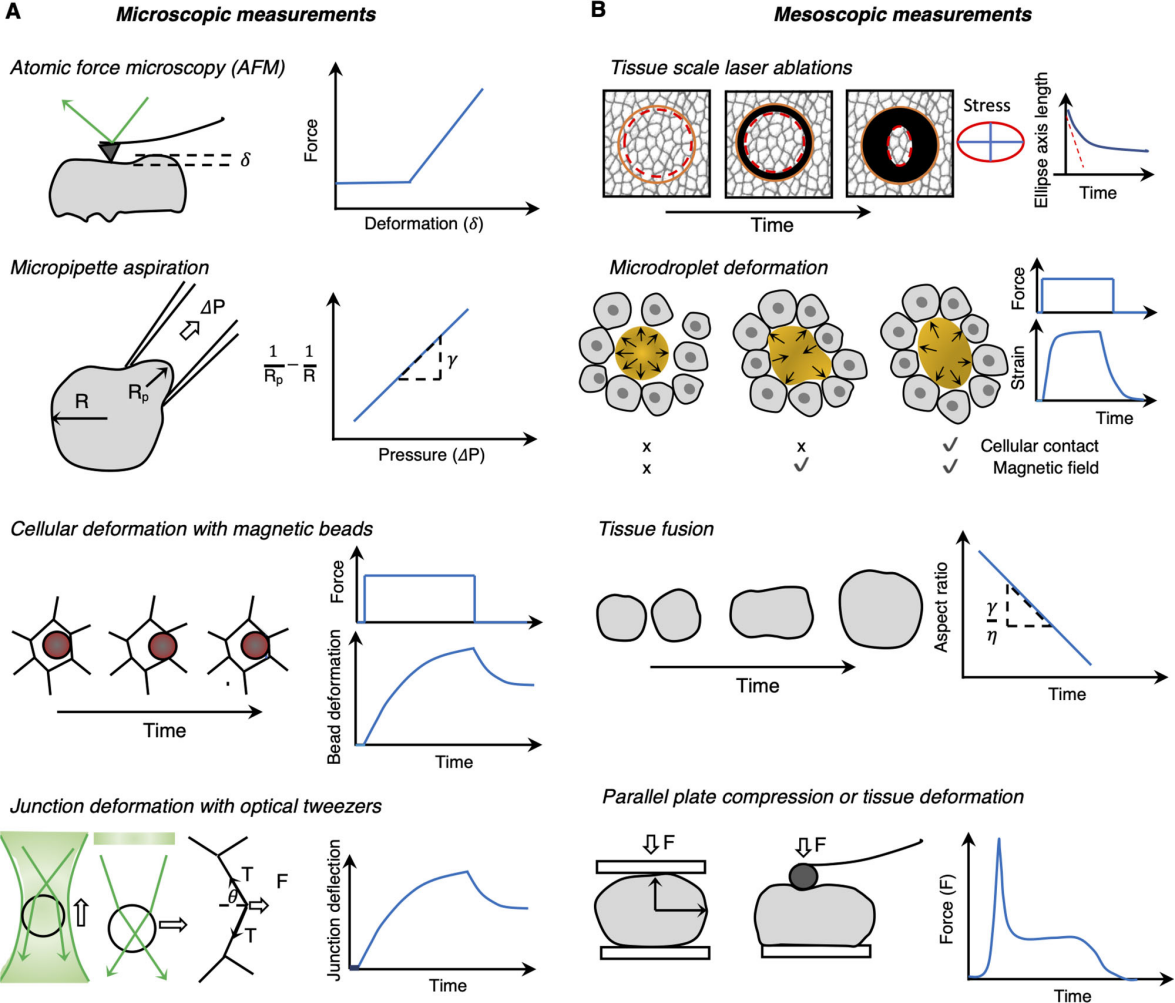
Rheological measurements (contact-based methods) to detect phase transitions in situ. **A** The class of microscopic techniques aim to probe rheology at the cellular scale where the measurements typically require a mechanical apparatus or the insertion of force probes and the determination of force-deformation curves. **B** Mesoscopic rheological measurements aim to measure supracellular properties relying on the same principle of utilizing a force-deformation curve but induce deformation at the multicellular level.

To some extent, the existence of the shape index as a hallmark of the solid state is reminiscent of a structural order parameter well defined for crystals. This suggests that though the material remains disordered, it is possible to recognize whether it behaves as a fluid or a solid by observing the shape of its constituents. One can argue that this is model-dependent and the only relevant approach to determine the states is to probe the mechanics (see the section “Experimental approaches and challenges to detect phase transitions in situ” and Fig. 5).

### Kinetic phase transition

Vicsek et al.^31^ first theoretically explored the emergence of self-ordered motion in systems of self-propelled particles. Their work, motivated by biological systems, modeled a system of particles moving with a constant absolute velocity and the direction of motion being the same as that of particles in its neighborhood of a given radius but with some random perturbation added (Fig. 3D). By performing numerical simulations, they observed that such simple interactions can lead to a novel phase transition from no transport to finite net transport through spontaneous symmetry breaking of the rotational symmetry, which is termed as kinetic phase transition. Conceptually such phase transitions can be seen as analogous to the alignment of spins in ferromagnetic materials where temperature serves the corresponding role as the random perturbations in the direction of motion for velocity alignment. While such transitions are not necessarily solid–fluid transitions, alignment of motion can facilitate collective motion and promote solidification^32^.

In general, such phase transitions are useful to understand collective cell motion within a cluster of cells depending upon the density and noise (i.e., the level of perturbations in the direction of motion) (Fig. 3C, D). In situations where density and noise are small, particles can form groups that move coherently in random directions; however, if the density is above a critical threshold then macroscopically ordered motion emerges where all particles tend to move spontaneously in the same direction (Fig. 3C, D). Biological evidence of such transitions have been observed in the collective migration of keratocytes of goldfish where interacting clusters of cells moving in groups can form near a critical density^33^.

Such transitions to an ordered state can be explained by short-range interactions without any explicit information about the knowledge of the directions of motion of neighbors. Similarly, velocity field of collectively migrating cells in a motile epithelium comprised of MDCK cells has been observed to be very coherent when plated at high cell density^34^. Recent work in carcinoma has shown that cells, upon overexpression of the small GTPase RAB5A, display collective motion reminiscent of kinetic (flocking) phase transitions. The persistent and coordinated movements in the tumor spheroids progressively remodels the extracellular matrix that further promotes collective invasion and dispersion of the carncinoma^35^. It is likely that the alignment of direction of motion of cells within a local neighborhood can be a result of chemotaxis or mechanotaxis^36^ that manifests in global alignment over several cell diameters.

### Dynamic modulation of cellular contacts/cellular fluctuations

Temperature is an important physical parameter relevant to phase transitions (Box  1) as it is a measure of the average kinetic energy of the constituent molecules. Consequently, molecules in solids move less as compared to the liquid or gas phase of the same material, effectively implying that the freezing point is at a lower temperature than the boiling point. Temperature is therefore well established as a control parameter for solid–fluid transitions and particularly for fluid-to solid “jamming” of foam-like systems from wet to dry states. It is only recently that an equivalent parameter termed as cell jiggling, has been proposed to be interpreted as the effective temperature^37,38^. Similar to the kinematic picture of the temperature, cell jiggling is a measure of the fluctuations in the contacts between cells and results from diverse molecular processes that can modulate cell–cell adhesion or cell contractility^39^. In this section, we propose several active processes within the tissue that can contribute to changes in cellular contacts/cellular fluctuations, and establish cell jiggling as an independent control parameter (independent of cell density and stress) for studying phase transitions in biological tissues.

#### Changes due to active tension fluctuations and jamming transitions

Solidification in multicellular organisms can be seen as the loss of cells’ ability to move and rearrange. Such process strongly depends on the active driven fluctuations that cells are able to generate. In zebrafish, a gradual solidification of cells underlies axis elongation^2^. In contrast to the chick, this process does not involve proliferation; instead mesenchymal cells from the mesodermal progenitor zone, which is located at the anterior of the animal, gradually lose their ability to rearrange as they move into the PSM (posterior). This fluid-to-solid transition is akin a jamming transition, cells becoming caged as they enter in PSM (Fig. 2B). Cells of the PSM form structures called somites that will give rise to the animal’s vertebrae. To map cell-scale mechanical properties, Mongera et al.^2^ used magnetically-deformable oil droplets that are injected in the tail of the zebrafish embryo. They measured the amount of stress needed to permanently deform the tissue (yield stress) and found that it increases in posterior-to-anterior direction indicating a more solid-like in the posterior and progressively solid-like in the anterior. In addition, the amplitude of tension fluctuations that govern the solid-to-fluid transitions appears to be tuned to enable the shaping of somite boundary, with the interior of the somite being solid while the immediately adjacent tissue is fluid (Fig. 2C)^40^. These observations are in agreement with a dynamic vertex model considering extracellular spaces and active tensions at cell–cell contacts^41^. Over a critical value of tension fluctuations, the model predicts the tissue behaves as a fluid. Below the critical value, a tissue can behave as a fluid or a solid depending on the developmental time. If the developmental timescale to form a structure is larger (smaller) than the stress relaxation timescale, the tissue behave as a fluid (solid)^41^. In a different scenario, glassy or jammed cell behavior during arrested coalescence of active drops was also reported through agent-based simulations of embryonic stem cells^42,43^.

#### Changes due to cell divisions and cell death

Cell divisions can often destabilize cell–cell contacts due to mitotic cell rounding. Tissue properties can be modulated depending upon the frequency of occurrence and synchronization of divisions of constituent cells. Together with cell divisions, cell death can introduce stress sources that, in general, are anisotropic and thus are able to create effective shear viscosity causing the tissue to behave as a viscoelastic fluid with a relaxation time set by the rates of division and apoptosis^26^. Both cell divisions and cell death in epithelial tissues can be a source of cellular rearrangements that locally fluidizes the tissue. In an early chick embryo, cell division drives cell intercalation events during gastrulation^25^ as discussed in the section “Changes due to actively driven cellular re-arrangements“. Junctional remodeling of dividing cells requires in their neighborhood low cortical actomyosin such that the dividing cells can deform and displace the neighbors. High turnover of the cortex results in low cortical rigidity and low E-cadherin junction stability. Increase in F-actin and Myosin stability impairs division-mediated rearrangements^25^. This requirement at the cellular level renders the tissue more fluid-like and enables large-scale flow patterns observed in gastrulating chick embryos.

Even in the case of non-epithelial tissues, cell division can be the driver for fluid-to-solid transitions (Fig. 4C). For example, in most early embryos before the activation of zygotic genome, cell divisions are synchronous and depending upon the species, rate of cell divisions will dictate the timescale of changes in tissue properties. In particular for zebrafish embryos, the first 2.5 h of development are accompanied by synchronous cell divisions almost every 15 min, effectively implying that all cells undergo mitotic cell rounding and loose cell–cell contact every 15 min. This clearly affects the overall mechanical properties of the tissue and its phase (solid-like or fluid-like) depending on whether or not the cells are in contact with each other.

As development proceeds, cell division can be a source of regionalized modulation of tissue properties as observed in the case of blastoderm spreading at the onset of zebrafish gastrulation by Petridou and colleagues^8^. They showed that cell rounding during divisions (cleavage cycle 12 and 13) in the central blastoderm makes the tissue more fluid-like. However, marginal cells activate noncanonical Wnt signaling (Wnt11-Fz7) locally and thus increase cell cohesion to counteract the effect of mitotic rounding on contact disassembly. The cohesion or solid-like behavior results from Wnt11-Fz7 signaling-dependent increase in actomyosin contractility which promotes E-cadherin-actin localization at cell–cell contact edges^44^. The authors further showed that such a spatially restricted change is tissue property is crucial for tissue morphogenesis since a uniform change to fluid-like behavior leads to reduced blastoderm thinning as seen in wnt11/slb-mutant embryos^45^.

In growing tissues, cells crowd, which inevitably results in dense packing and potentially solidification. In epithelial tissues, cell delamination, a process that precedes cell death was shown to counterbalance overcrowding^46^. Cells delaminate by loss of cell–cell junctions and reduction of apical area, before cell neighbors squeeze them out (Fig. 4D). As previously suggested theoretically^47,48^, crowding could mechanically feedback on cells to buffer tissue growth and ensure tissue homeostasis. For instance, a form of inhibition of cell proliferation known as contact inhibition has been shown to result from changes in density^49^. Impairment of the mechanical feedback between crowding and growth may favor hyperplasia and tumor formation, which is often accompanied by a solidification process.

#### Changes due to cortical pulses and waves

Pulsed contractions of cells have been shown in early mouse embryos^50^ to manifest as periodic cortical waves on a short timescale when cells are disengaged from adhesive contacts. However, in tissues, such cell-autonomous pulsed contractions, when confined or suppressed, due to Cadherin-mediated cell–cell contacts get redirected away from cell junction and thus result in tissue-level forces at the long timescales. Particularly in case of early (eight-cell stage) mouse embryo, such actomyosin contractility results in compaction, which increases the overall mechanical integrity of the embryos^50^. Such pulsed contractions of an actin–myosin network have also been shown to drive cellular shape changes in *Drosophila*^51–54^ and result in overall tissue flow due to the viscoelasticity of cells and tissues that allows them to deform permanently despite transient forces^9^. Overall, it is reasonable to speculate that a balance between cell adhesion and cell-autonomous pulsation can result in long-term forces in multicellular systems that can influence solid-like or fluid-like behavior of the tissue through dynamic modulation of cellular contacts/cellular fluctuations.

#### Changes due to cell–cell connectivity, percolation, and network phase transitions

Alterations in the connectivity of links in a network can lead to macroscopic changes in rigidity. The extent of connectivity can be seen in analogy to the ease of liquid percolating through a porous substance: higher connectivity implies absence of free passage for liquid to percolate through. This situation is termed as “percolation of rigidity” (instead of liquid) through the network. Similar to this well-studied process in physics, changes in cell–cell connectivity can alter tissue rigidity i.e., may lead to percolation. In an infinite network, percolation theory predicts a phase transition in rigidity above a critical density of links (Box  2 and ref. ^55^). For finite-size multicellular systems such as tissues, it was not shown if an abrupt change in viscosity can occur when stress propagates over the system size, when cell connectivity reaches a threshold. However, a recent study in zebrafish blastoderm demonstrated a genuine rigidity phase transition that results from changes in adhesion-mediated cell connectivity (Fig. 4F and ref. ^56^). The authors showed that when the average contacts per cell is low, the tissue, unsurprisingly, behaves a fluid but above a critical threshold of connectivity giant rigid clusters (Box  2) abruptly emerge and span the whole network to resist deformation, thereby rendering the tissue behavior more solid-like. Through a combination of genetic perturbations to alter cell connectivity (either by reducing the levels of E-cadherin expression or by downregulating Wnt/PCP pathway (Fig. 4C) or by altering cell fate specification) it was shown that cell connectivity is a reliable parameter to predict changes in tissue viscosity using rigidity percolation analysis. Such network phase transitions are another example of how biological systems utilize phase transitions for sculpting tissues during morphogenesis^57^). On the contrary being close to criticality can lead to instability which is not typically observed in biological systems^56^ and we envision that future studies will address this conundrum opening new avenues in our understanding of the role of phase transitions in biological systems.

Box 2 Box definitions 2 **Symmetry and symmetry breaking** The symmetry (geometric) of an object refers to its invariance under geometric transformations. For example, an object is rotationally symmetric if it can be mapped onto itself after rotation. Symmetry breaking is a process, usually induced by small fluctuations, that brings a system from a symmetric to a less symmetric state. The final state is different from the initial state and more features are needed to describe it. For example, liquid water looks the same in all directions. When it changes into a snowflake, it loses rotational symmetry and looks the same in only six directions.

**Glass transition and jamming transition** The glass transition refers to a transition from a viscous liquid to an amorphous (disordered, non-crystalline) solid. Jamming transition is a glass transition at zero temperature used to denote a transition from a liquid or “floppy” state to a rigid state. It covers situations as different as the formation of traffic jams or the piling up of sand or sugar grains or the glass transition. It is accompanied by a drastic drop of the motility of the constituents. It can thus be considered as a kinetic phase transition. Jamming transition is a generic phenomenon that manifests in the “freezing” of the system. For example, when a given volume of sand is poured onto a table, the flow stops spontaneously and a mechanically stable pile is obtained. A colloidal assembly shows the same type of transition when the volume fraction of colloids is large: in a system of hard spheres at high concentration, particles are trapped in transient cages formed by their neighbors. Close to jamming, it is the rearrangement of their cage that governs the (slow) dynamics^105^ and resistance to deformation. The slowing down of movements is associated to clusters of particles, whose characteristic size increases with crowding^106^. A striking property of jamming systems is that a modest change in control parameter, (e.g., increase in density) leads to the divergence of the effective viscosity and the emergence of a finite rigidity. For a detailed review of jamming and its sources in biological context see ref. ^20^.

**Percolation and network phase transitions** In a network addition or removal of nodes and/or links can change the macroscopic behavior of the network. Below a critical density of links, the network can be collapsed into smaller connected clusters owing to increase in the number of continuous deformations within the network. Alternatively, above this critical density of links, the network will behave as rigid, or in other words rigidity *percolates* through the system^55,107–110^. This change in the structure of the network leads to a phase transition: below the critical density, the network will have large floppy regions with a few rigid inclusions whereas above this critical value, a rigid cluster of nodes that can span the whole network, termed as a *Giant Rigid Cluster*, emerges abruptly^56,110,111^. Percolation theory predicts that this critical value is two-thirds of the maximal average number of contacts.

## Experimental approaches and challenges to detect phase transitions in situ

Solid to fluid transitions in inert materials are accompanied by abrupt changes in the long-range order of the constituent particles. Yet biological materials, as discussed before, remain largely disordered. This poses a challenge to recognize whether it behaves as a fluid or a solid as no overt properties can conclusively establish the phase of the material and thus the occurrence of a transition. However, a salient feature of phase transitions is the drastic changes of material properties. For instance, jamming is accompanied by the divergence of the viscosity and the acquisition of a finite rigidity. Therefore an obvious approach to detect such phase transitions in a tissue or cell collective, is to mechanically probe it and measure the rheological properties in situ. Such class of measurements can be termed as rheological measurements.

Another approach to infer the phase of the tissue by focussing on the dynamics i.e., imaging and tracking cell behaviors and cell shape. Shape of constituent cells can provide hints of the mechanical state of the tissue. Analyses of trajectories of single cells or cell clusters and their diffusion properties are informative of phase properties. For example, fluids are characterized by particles diffusing freely and being able to pass each other under shear, while glasses (jammed solid) show cage effects that limit the diffusion of particles. Such types of measurements can be termed as kinematic measurements.

### Rheological measurements (contact-based methods)

Rheological experiments are classical in material sciences and consist in measuring the flow and deformation of a material under applied force. Their implementation in biological systems is often challenging but recent years have seen both the adaptation of established techniques as well as the development of new ones (for a detailed review see ref. ^58^). These types of measurements typically require a mechanical apparatus or the insertion of force probes and the determination of force-deformation curves (Fig. 5). Depending upon the scale of measurements, we can classify those as microscopic (that measure rheology at cellular scale) or mesoscopic measurements (at the supracellular or tissue scale).

The class of microscopic measurements includes approaches such as atomic force microscopy (AFM)^10^, micropipette aspiration^59^, optical tweezers^60^, cell vertex displacements after subcellular laser ablations^52^ and magnetic beads, all of which rely on a force-displacement (deformation) curve based inference (Fig. 5A). Another emerging method is Brillouin microscopy (for a detailed review see ref. ^61^) that is a label- and contact-free method to measure viscoelastic properties of tissues. Classic examples of the usage of some of these techniques (for a more detailed list, see ref. ^8^) include single-cell force spectroscopy (SCFS,^62^) to measure adhesion of germ-layer progenitors in zebrafish embryos^10^, fusion experiments to infer tissue surface tension differences^63^, AFM to measure elasticity of Xenopus head mesoderm^64^ or viscoelasticity of endothelial, cardiac muscle and skeletal muscle^65^, optical tweezers to deform cell junction and viscous dissipation in the early *Drosophila* epithelium^9,66^, Brillouin microscopy to map elastic properties of normal and diseased human corneas^67^ or ECM stiffness in zebrafish notochord^68^ and micropipette aspiration to measure tissue viscoelasticity in different vertebrate embryos: zebrafish blastula^8^, Xenopus^69^, chicken^70^, and mouse^50^.

Mesoscopic rheological measurements aim to measure supracellular properties relying on the same principle of utilizing a force-deformation curve but induce deformation at the multicellular level (Fig. 5B). Tissue-scale laser ablations can probe anisotropy in wound closure^71^. Parallel plate compression techniques draw on the classic compression techniques used in material science but at microscopic scale where surface tension effects dominate tissue deformation and shape^72^. Microdroplet deformations can measure stiffness anisotropy^73^ and tissue mechanical response to large deformations can be studied using ferrofluid drops under uniform magnetic field^74^. For instance, Mongera et al.^2^ injected such magnetically-deformable oil droplets in the tail of the zebrafish embryo and measured that the amount of stress needed to permanently deform the tissue (yield stress) increases in posterior-to-anterior direction along the body axis indicating a more fluid-like in the anterior and progressively solid-like in the posterior. Soft elastic microspheres in alginate or polyacrylamide can be used to measure both isotropic (tensile and compression) and shear stresses^75,76^. Techniques such as AFM have also been adopted for supracellular measurements by attaching a large bead to the AFM cantilever to measure tissue-scale deformation^64^.

In both classes of measurements, in order to detect evidence of phase transitions, it is crucial to perturb and probe the tissue in a native state as possible and preferably infer the dynamic state of the tissue rather than a passive measurement of mechanical properties of its constituent cells. However, this is not always possible and thus requires a prudent selection of technique and the analysis method for a given system because the inferred property can have large variation depending upon the magnitude and rate of applied force, geometry and configuration of the probe used and the region of the tissue probed (for a detailed review see ref. ^77^).

### Kinematic measurements (non-contact methods)

**Fig. 6:**
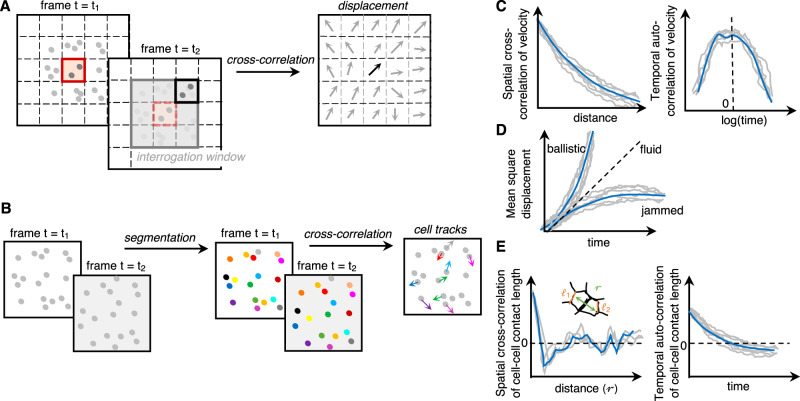
Kinematic measurements (non-contact methods) to detect phase transitions in situ. **A**, **B** Velocity measurements can be done either based on the movement of a collection of particles (global flow patterns) by particle image velocimetry or related techniques (**A**) or the movement of individual particles through segmentation and tracking (**B**). **C** Higher-order measures on velocity such as spatial cross-correlation and temporal autocorrelation allow inference of relaxation times and correlation lengths for collective cell movement respectively, both of which are useful to detect phase (jamming) transitions in cell monolayers (adapted from refs. ^4,78^ with permission from the *Proceedings of the National Academy of Sciences of the United States of America* and *Soft Matter*). **D** Root-mean-square displacement can be inferred from individual tracks of the particles (cells) and can be used to infer caged vs uncaged dynamics. **E** Higher-order measures can be made on cell–cell contact length (*l*) in a segmented image: spatial cross-correlation of cell–cell contact lengths separated by a distance *r* or temporal autocorrelation of cell–cell contact length are useful to detect the amount of “jiggling” characteristic of phase (jamming) transition (adapted from ref. ^2^ with permission from *Nature*).

This class of measurements relies on analyses of movements, shapes of the cells in the native environment to gain insights into the mechanical state of the tissue (Fig. 6). Live imaging, fluorescence or otherwise, is a crucial tool for these measurements.

For example, velocity measurements and higher-order measures, such as velocity spatial correlation and velocity temporal correlation, are useful to detect phase transitions^4^. Velocity auto-correlations give access to relaxation times^78^ which are expected to increase dramatically close to glass transition. Velocity spatial correlation is able to capture collective movements and their correlation lengths. These measurements have been applied to characterize collective movements and jamming transition in cell monolayers^79^. We foresee that they will be efficiently applied in vivo and in three-dimensional systems, as the approach is non-invasive and relies on imaging.

## Going beyond phase transitions: slow changes to tissue stiffness

Changes in material property of biological tissues can happen gradually on the timescale of several hours to days without the presence of any critical transition point, typical of phase transitions. Despite the slow pace, changes in mechanical properties can be dramatic. Solidification or fluidization of tissues in such cases can result from changes in both intracellular composition and extracellular environment.

### Changes due to cellular composition

**Fig. 7:**
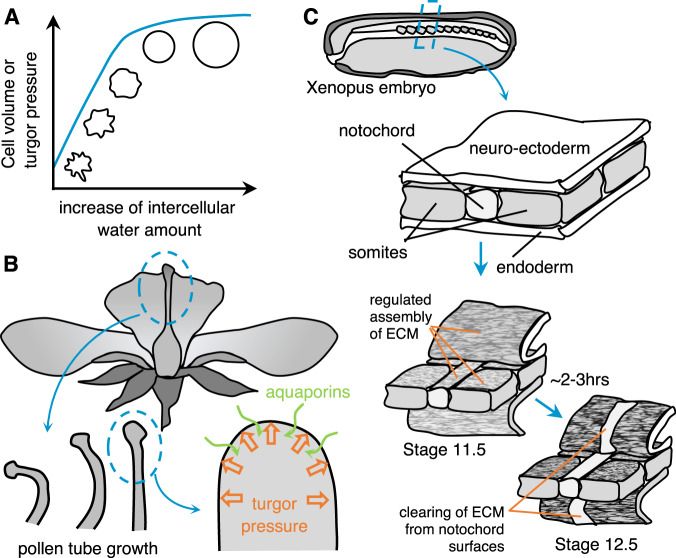
Changes to tissue stiffness resulting from changes in cellular composition and extracellular environments. **A**, **B** Amount of water present in plant cells, builds up the turgor pressure (**A**), and drives local morphogenesis and overall integrity and rigidity of the tissue, such as the growth of the pollen tube in flowers (**B**, adapted from ref. ^80^ with permission from the *Annals of Botany*). **C** ECM components such as fibronectin fibrils delimit tissue boundaries, such as between neural/mesoderm interface in developing Xenopus embryos (adapted from ref. ^87^ with permission from *Developmental Dynamics*).

Amount of water present in a cell is a crucial factor determining the mechanical properties of the cells. Particularly in the case of plant cells, that possess cell walls, hydrostatic pressure within cells due to influx/outflux of water can reach as high as 20 atmospheres (~2 MPa,^80^). This pressure, termed as turgor pressure, is thus crucial for driving local morphogenesis and the overall integrity and rigidity of the tissue. Unlike animal tissue where the cells are motile, plant tissues control their hardness by regulating the material properties of the cell wall and turgor pressure and can consequently lead to large-scale deformations of their structures. While the evidence for an abrupt phase transition resulting from such changes in the cellular composition is not yet known, modulating the amount of water is still a robust mechanism to control overall tissue rigidity. For instance processes in flower development such as anther opening for the release mature pollen (process called anther dehiscence^81,82^), or opening of floral buds due to expansion of petals that also exposes the stigma on carpels for pollination^83^ both result from a sequence of hydration and dehydration of specific cell populations that spatially regulates the relative stiffness and thus leads to controlled overall deformation of the tissue (Fig. 7A, B). At the molecular level, the inflow and outflow of water are controlled by spatial regulation of the distribution of K+/Na+ ion transporters and aquaporins. Regulation of water permeation has recently been reported as a mechanism to control cell migration in confined microenvironments^84^. Stroka et al. showed that confined tumor cells establish a polarized distribution of Na+/H+ pumps and aquaporins in the cell membrane, to create a gradient of water and ion flow between the leading and trailing edge of the cells and therefore achieve net cell displacement.

### Changes due to extracellular environments

Population-level behavior of cells as solid-like or liquid-like can also be determined by factors extrinsic to the cells. In this regard, the extracellular matrix (ECM) is a key determining factor that is secreted by the cells and in turn influences the collective cell dynamics through mechanochemical control of cell behaviors. The changes in overall tissue stiffness can be a direct outcome of the amount and orientation of the secreted ECM. Bone and cartilage tissues are classic examples where soft aggregates of mesenchymal cells differentiate into large hypertrophic chondrocytes that synthesize collagen type X and calcify the ECM around them^85^. Subsequently, the coordinated apoptosis of such chondrocytes along with their replacement with osteoblasts that further secrete collagen type I, changes the overall stiffness of the tissue to form rigid bone structures (for review, see ref. ^85^). In diseases such as osteoarthritis, osteoblasts do not secrete sufficient ECM, thereby affecting the mechanical properties of the bone. Another striking example is the secreted chitin matrix that, in combined with calcium carbonate, becomes a solid material that makes the exoskeleton in the case of arthropods such as the shell of crustaceans and molluscs^86^).

The formation of solid structures such as bones, cartilages, and exoskeletons is gradual and spans several days. Effect of matrix deposition at smaller timescales and its influence has been widely studied in the development and diseases such as cancer. One of the first evidence of such effects in development was observed in the Xenopus embryo^87^. The extension of the body axis along the A/P axis is accompanied by a stiffening of the dorsal involution margin. Stiffening was shown to be larger along the A/P axis than along the mediolateral axis. Tissue stiffening was proposed to arise from either the reinforcement of the cytoskeleton or the deposition of extracellular matrix, which would contribute to the maintenance of a rigid axis^88^. There is evidence that fibronectin fibrils are elaborated at tissue boundaries (Fig. 7C)^87^. Their pattern could control mechanical properties such as the elastic modulus, but also serves as lubrication reducing shear between tissues during morphogenesis.

More generally, changes in the extracellular environment are known to affect tissue differentiation^89^, orientation, and movement all of which can contribute to tissue stiffening. For instance, it was shown recently that in *Drosophila* follicle, basement membrane stiffness, sensed via Src tyrosine kinase alters junctional E-cadherin trafficking of cells at the follicle anterior^90^. Consequently, without any changes in average cell shape or oriented cell division, cells get reoriented in an edgeless tubular epithelium primarily due to the orientation of the extracellular matrix thereby controlling the shape and stiffness of the three-dimensional tissue. Another scenario of ECM controlling cell movement is the previously discussed case of graded motility of cells in the presomitic mesoderm (PSM) of chick embryos^21^. Herein, the gas-like behavior of mesenchymal cells (resulting in non-directional Brownian motion) at the individual cell level is converted to a graded flow pattern resembling a liquid due to the movement of the underlying extracellular matrix.

There is a large literature on how ECM components and biophysical properties induce EMT and promote migratory behaviors (for a review see refs. ^91,92^). Whether tumor growth and spreading is akin to a phase transition has been a debated question for a decade (e.g., ref. ^93^). There are striking similarities between processes in development and cancer, regarding their sensitivity to ECM stiffness, and more generally to the stiffness of the environment. ECM stiffness might promote tumor growth, spreading, and metastasis along gradients of ECM rigidity, a process termed durotaxis^94^: similarly, substrate stiffness can trigger collective cell migration by promoting EMT during neural crest formation in vivo^64^. In the latter case, cells of the neural crest migrate as a response to the stiffening of the supporting mesoderm. Tissue fluid-like or solid-like behavior is thus regulated by intrinsic and extrinsic factors.

Usually, instances of solidification such as these are gradually occurring on the timescale of several days without any critical transition point typical of phase transitions.

During morphogenesis, tissues are subjected to external forces and mechanical constraints by the surrounding tissues and environment (extracellular matrix, shells, perivitelline membranes). Several groups have started to address the question of how tissues respond mechanically to such forces and constraints. Recent reports suggest that mechanical properties, such as stiffening are induced by external conditions. Stretch-induced stiffening has been proposed as a mechanism to limit changes in shape after deformation. Using a stretching device, Duda et al.^95^ applied external load on the *Drosophila* wing disk epithelium and observed a mechanical response of the tissue, that formed polarized actomyosin cables along the direction of stretch. Actomyosin networks sense and respond to geometrical and mechanical constrains by adopting different configurations^96^. They can form rings or asters, which in turn, could orient force generation and/or modify mechanical properties. Laser incisions or cauterization performed at different positions altering the boundary are a means to determine the contributions of external tissues to mechanical properties^52,96,97^. It remains to be shown whether modulation of external conditions could lead to phase transitions. Strain-stiffening could be viewed as a general mechanism of solidification that reduces or even prevents tissue deformation.

## Limitations of analogy between living and non-living systems and future directions

The analogy between collective effects in living systems and the well-known phase transitions in inert systems has persuasive power. For example, adapting the classical three-axis jamming phase diagram to the case of cell monolayers was instrumental to highlight key parameters that may control fluid-to-solid transitions in tissues, namely cell motility/cell jiggling, the inverse of cell–cell adhesion and cell density as counterparts of temperature, stress and volume fraction or density^37^. However, the uniqueness of the parameters is questionable. In confluent monolayers in which the volume fraction is close to 1, this parameter cannot be relevant to describe the jamming transition^98^ and instead a parameter featuring the shape of the cells was proposed^17^. This exemplifies the necessity to search for control parameters and test their relevance.

Similarly, while it is clear that fluctuations will in general contribute to the fluidization of the tissue, it is crucial to probe the exact nature of these fluctuations (thermal or nonthermal). It can be misleading to draw an analogy between cellular motility and (effective) temperature because the actively driven fluctuations that cause cellular movement and thermal fluctuations are very different in nature^99–101^. Actively driven stresses lead to qualitatively different steady states than those given by the Boltzmann distribution that prevails for thermodynamic systems. This hinders the definition of an effective temperature for living systems^102^. The active fluctuations generated by molecular motors determine the characteristic time of shape fluctuations, which in turn lead to cellular rearrangements and cellular motility. We know little about the relationships between the molecular and cellular scales, and it will be important to develop physical models and theories that could link them, which will help us to define effective parameters controlling phase transitions. The application of the concept of phase transitions to biological systems is limited by the out-of-equilibrium nature of the living. The observed states of living systems are not equilibrium states, but rather transient or steady states that emerge from nonequilibrium dynamics. During embryogenesis, the succession of states that arise from changes in gene expression, dynamic adhesion, and cytoskeletal structures may render the identification of phase transitions risky or even misleading. Nevertheless, characterizing the material properties of such states remains essential and we believe that in some cases they will reveal transitions in behavior, some of which will be the hallmark of phase transitions. The location of the transitions will depend on the specifics of the biological systems, but, as it has been shown for other dynamic systems^103^, it is likely that features insensitive to details will emerge.

An appealing but daunting task is to relate the physical parameters that control the phase transitions to biological entities. There is an inherent risk in trying to match a protein function to a physical parameter, as many proteins contribute to multiple physical properties. For example, myosin-II activity enhances cell fluctuations but is also able to change cell stiffness as an actin cross-linker. Keeping this in mind, using acute genetic and molecular perturbations combined with mechanical measurements and imaging will be essential to decipher important players and determine where the system sits in the phase diagram, and hopefully explore the parameter space.

Biological systems exhibit a strong coupling between the timescales of changes in mechanical properties and the timescale of deformations, as a result of which stresses can play a very different role in phase transitions, jamming in particular, when compared to inert materials. Feedback is at play between mechanical and biochemical signals from the molecular to the tissue scales. In addition, size and geometry can play a critical role on the collective behaviors of multicellular systems. This makes living systems very special with respect to inert systems.

Future work should provide insight into the multiscale coupling of geometry, mechanics, and biochemistry and how they integrate to define the collective behaviors of multicellular systems, and the parameters that control phase transitions during the formation of organisms.
